# Reliability Assessment of High-Speed Train Gearbox Based on Digital Twin and WHO-WPHM

**DOI:** 10.3390/s25206418

**Published:** 2025-10-17

**Authors:** Tengfei Wang, Yun Chen, Siying Li, Jinhe Lv, Yumei Liu, Jinyu Yang, Qiushi Yan

**Affiliations:** 1 School of Civil Engineering and Transportation, Beihua University, Jilin 132013, China; wtf7826@163.com (T.W.); 18649118323@163.com (S.L.); lvjinhe_1987@163.com (J.L.); 159313522933@163.com (J.Y.); yanqiushi2014@163.com (Q.Y.); 2 Transportation College, Jilin University, Changchun 130022, China; lymlls@163.com

**Keywords:** digital twin, high-speed train gearbox, wild Horse optimizer, Weibull proportional hazards model, local tangent space alignment, reliability assessment

## Abstract

The gearbox is essential for power transmission in high-speed trains, and its reliability directly impacts operational safety. Accurate monitoring data and effective assessment methods are crucial for accurately assessing its reliability. This study is based on digital twin (DT) technology, precisely deploying virtual sensors to collect vibration data from critical measurement points accurately. By integrating the Wild Horse Optimizer (WHO) and the Weibull Proportional Hazards Model (WPHM), it achieved reliability assessment for a high-speed train gearbox. First, a DT framework for the high-speed train gearbox was established. Taking the gear pair, a critical power transmission component in the gearbox, as an example, a DT model of the gear pair was built on Ansys Twin Builder, virtual sensors were deployed at critical measurement points, and vibration acceleration data was collected. Then, a WPHM reliability assessment model was established, and the WHO was used to estimate and optimize the WPHM parameters. Finally, the response covariates reduced by the Local Tangent Space Alignment (LTSA) were used as model inputs, and the WPHM was applied to assess the reliability of critical parts based on the collected data. The web-deployed DT model was delivered within 13 s. This achieved a simulation acceleration factor of 2.35 × 10^4^, compared to traditional methods. The number of iterations for the WOA was reduced by 62.9% compared to the WHO and by 48.1% compared to the HHO. The reliability assessment results align with the actual operating mileage status of the gear pair, thus validating the effectiveness and feasibility of this method.

## 1. Introduction

**Figure 1:**
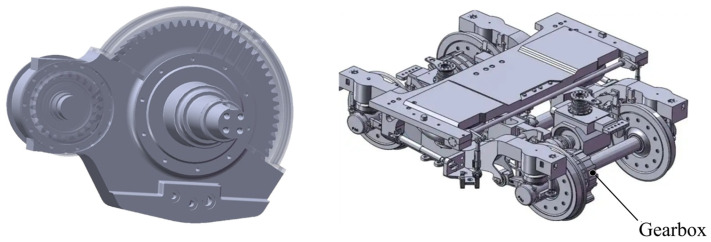
High-speed train gearbox.

The gearbox is a critical component in transmitting torque and driving high-speed trains. Its reliability directly determines the safety and reliability of high-speed train operation. Their structure and installation location are shown in Figure 1. Accurately understanding the reliability of key components of a high-speed train gearbox during operation is an important guarantee for the continuous and stable transmission of power under high-speed, high-torque alternating load conditions. This is of great significance for ensuring the stable operation and efficient maintenance of high-speed trains.

The foundation for assessing the reliability of mechanical components is obtaining real-time, accurate monitoring data. Currently, the main methods for obtaining data include contact-based detection using physical sensors and non-contact detection using information transmission methods such as light, sound, and magnetism. In recent years, the development of DT technology has provided new methods for mechanical detection. DT technology uses digital means to create multidimensional virtual models of physical entities and can be used to dynamically simulate, map, predict, and control physical entities in the real world. In the field of rail transit, Wu et al. [1] applied the seven-dimensional DT framework to high-speed train bogies, resolving the challenge of real-time online monitoring for bogies. Liu et al. [2] established a structural simulation model for high-speed train bogies through finite element modeling and mechanical analysis. They developed a lightweight agent model that connects input data with a DT display platform, enabling real-time output and rapid prediction of dynamic performance and structural damage status, thereby meeting the real-time requirements of the DT. Liu et al. [3] employed a specific aircraft engine bearing as a case study and selected prognostic features from the physical, virtual, and service layers of its DT as inputs for fault prediction. Under comparable convergence rates, this model achieved significantly lower prediction error than conventional methods. Hu et al. [4] developed a DT system for aircraft engine gears with the help of the Shanhai Whale Visualization software. The system achieved an overall accuracy rate of 96.3% in condition monitoring by detecting vibration signal changes through piezoelectric sensors and using machine learning to identify gear failure modes. Xia et al. [5] established a DT model of a truck transmission capable of generating vibration data under different health conditions. This effectively improved the performance of fault diagnosis when there was little fault measurement data available. In the study of critical gearbox components, numerous publications have explored the extensive application of DT in performance degradation and wear assessment. Feng et al. [6] integrated a gear transmission dynamics model with a degradation model to establish a DT model, enabling the investigation of dynamic response characteristics during gear wear processes and achieving an average tooth flank degradation assessment accuracy of 92.71%. Matania et al. [7] applied Paris’s law to simulate gear root crack propagation in a planetary gear DT system and estimated fault severity and remaining time before repair under zero-fault-triggered learning. Xu et al. [8] established a DT dynamic model based on first-principles calculations and combined it with a deep reinforcement learning model to construct an autonomous learning strategy update and iteration external gear DT model. Based on the model, they conducted a qualitative analysis of the wear degree of the gear pump and verified the feasibility and effectiveness of the model. Yang et al. [9] proposed a DT framework based on meta-behavioral theory and used the particle filter algorithm to combine data-driven and model-based modeling methods to establish a new DT drive prediction model. The minimum prediction error of the CNCMT turntable transmission unit life degradation was 3.17%. To achieve virtual–physical interaction and visualization analysis for DT, Li et al. [10] used Unity3D virtual engine technology to construct a DT model, collected data such as vibration and temperature, and used a data-driven engine to achieve synchronous simulation between the DT model and the gear test bench, thereby obtaining the time-domain and frequency-domain changes in the simulated vibration. Xi et al. [11] proposed a new method for three-dimensional (3D) gear pitting detection. Combining the gear pitting model and Unity, a virtual fringe projection profilometry system was established in a DT virtual environment, which realized the generation and phase recovery of deformed fringe patterns. The system was then applied to enhance the training samples of the gear pitting detection network and improve the accuracy of gear pitting detection. Wang et al. [12] established a planetary gear fault diagnosis model based on empirical mode decomposition and achieved accurate behavior mapping with the DT created on Unity3D, resulting in a gear fault diagnosis accuracy rate of 94%. Xu et al. [13] proposed a remote intelligent maintenance framework for wind turbines based on DT, applying Visual Studio 2019 and other platforms to integrate the WOA-TCN-Attention computing model, thereby solving the problem of difficult real-time fault prediction.

In the reliability assessment of gearbox systems and critical components, the current effective approach involves evaluating reliability based on collected vibration signals, combined with mechanism model-driven, probability model-driven, and data-driven methodologies. Specifically, mechanism model-driven approaches do not rely on extensive historical data; instead, they utilize mathematical–physical equations to describe the system’s dynamic characteristics and the behavior of performance degradation processes. Wu et al. [14] established a rigid-flexible coupled dynamic model of a high-speed train gearbox using Simpack software and the Weber/Banaschek method. They analyzed the dynamic response and fatigue damage of the gearbox under wheel plane and wheel polygon excitation, confirming that the impact of gearbox fatigue damage increases with rising vehicle operating speeds. Zhang et al. [15] combined fault mechanism modeling with gradient-boosting decision tree condition diagnosis to evaluate harmonic gear transmission system reliability, demonstrating a 33% reduction in relative error during validation. Probability model-driven approach, the performance degradation process of components or systems is treated as a stochastic process that evolves over time. Xia et al. [16] established a mixed Copula model using Gumbel and related functions and optimized its weight coefficients to enable efficient failure probability estimation for gear subsystems with limited sample data. This approach enhanced both computational efficiency and reliability prediction capabilities. Liu et al. [17] established a reliability model integrating Gamma processes, homogeneous Poisson processes, and mixed Copula functions. They quantified fault correlations using a Gumbel–Clayton–Frank hybrid Copula with multi-layer nested algorithms, validating the model’s consistency with operational conditions via planetary gear system experiments. Gao et al. [18] predicted the dynamic reliability of high-speed train gears under a single failure mode based on Gamma process theory and wear interference models. Using Copula functions, they captured the dependency relationships between gear pairs under different failure modes and evaluated their joint reliability under multiple failure models. Liu et al. [19] focused their research on critical parts of a high-speed train gearbox. They used the Gamma process to describe part strength degradation and introduced Copula functions to establish a fault correlation model for gearbox systems. The evaluation results met engineering requirements. Data-driven approaches extract information and uncover patterns directly from data without relying on prior physical mechanisms or human experience. Gao et al. [20] proposed an adaptive maximum correlation kurtosis deconvolution hybrid model based on intrinsic time scale decomposition to extract bearing vibration characteristics and established a reliability model through a logistic regression model to obtain a reliability curve that is closer to reality. Zhang et al. [21] employed a radial basis function neural network to establish a vibration signal-based feature mapping model for system state characterization. Using the first principal component post-dimensionality reduction as a rolling bearing degradation indicator, they conducted reliability assessment via a WPHM, with results consistent with full life-cycle testing. Dai et al. [22] proposed a Wavelet Kernel Network–Bidirectional Gated Recurrent Unit hybrid neural network for health index construction, integrating Wiener processes to represent non-monotonic health indices and effectively extract deep vibration features for reliability assessment. Gao et al. [23] processed multidimensional time-frequency and time-domain bearing features using isometric mapping, characterized bearing degradation status via comprehensive feature indicators, and modeled rolling bearing reliability with logistic regression, validated against University of Cincinnati bearing life test data.

The aforementioned scholars had conducted in-depth explorations into the application of DT technology in mechanical inspection. However, existing methods for constructing DT models of mechanical structures still relied on high-computational-power equipment, requiring substantial time for modeling and involving complex computational processes. In the field of data acquisition for mechanical reliability assessment, most existing studies relied on physical sensors to collect vibration data in order to establish reliability and degradation models. However, due to the structural constraints of the components under test, precisely positioning measurement points at critical locations was challenging. This resulted in inaccurate data collection and compromised the reliability of the assessment. In terms of assessment models, existing reliability models suffered from complex construction and solution processes, lacked clear physical explanations, and exhibited poor interpretability. This study referenced the five-dimensional DT system framework to create a high-fidelity DT model of a high-speed train gearbox on the Ansys Twin Builder. Leveraging the platform’s dynamic model reduction, it significantly reduced modeling time and computational load for the DT model. This effectively addressed the current limitations in real-time performance of DT models and enhanced their engineering implementation feasibility. By deploying virtual sensors within the DT model, precise operational data from critical locations on rotating components could be directly obtained. This overcame the limitations of traditional inspection techniques, which were constrained by mechanical structures and could only place measurement points on the gearbox housing. In terms of reliability assessment, this study integrated the WHO and the WPHM to achieve rapid convergence and global optimization of the estimated parameters. This approach effectively addressed the issues of complex parameter estimation processes and limited accuracy in traditional WPHM methods. Ultimately, the accuracy of high-speed train gearbox reliability assessment was enhanced through two dimensions: monitoring data and evaluation models.

The remainder of this paper was organized as follows: Section 2 introduced the framework and related theories for establishing a DT model of a high-speed train gearbox. Section 3 constructed a gearbox reliability assessment model integrating WHO and WPHM. Section 4 used a gear pair in a high-speed train gearbox as an example to establish a gear pair DT model on the Ansys Twin Builder and completed model verification, data collection, and DT model deployment. Section 5 evaluated the reliability of the high-speed train gearbox based on an optimized WPHM. Section 6 presented the conclusions of this study.

## 2. DT Modeling Framework and Theoretical Foundations for High-Speed Train Gearbox Systems

### 2.1. DT Modeling Framework

**Figure 2:**
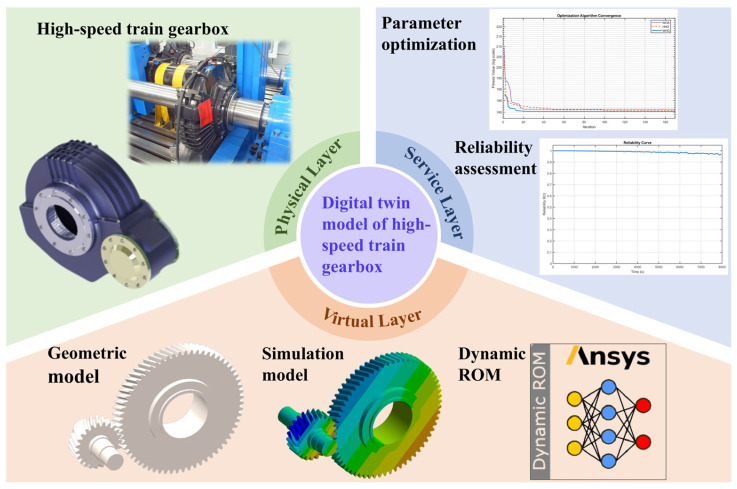
DT framework for high-speed train gearbox gear pair.

The DT model described in this article was constructed and deployed using SolidWorks 2019, Ansys Workbench 2021 R1, Ansys Twin Builder 2022 R1, Ansys Twin Deployer 2022 R1, MATLAB R2024b, and other software. The critical components of a high-speed train gearbox include gear pairs, bearings, and the gearbox housing. As the gear transmission mechanism is the primary component for power transmission in the gearbox and operates at high speeds while transmitting large torques, it is difficult to collect operational data from gear surfaces. Therefore, the DT model of a high-speed train gearbox, which is established and deployed in this paper, uses gear pairs as an example. Figure 2 shows the DT framework outlined in this paper, which comprises the physical layer, virtual layer, and service layer.

The physical layer constitutes the foundation of the DT framework proposed herein, serving as the cornerstone for virtual model construction, data acquisition, bidirectional data interaction, and reliability assessment services. Key parameters within this layer encompass real-time operational loads, geometric parameters, material properties, and sensor configurations of the gear pair.

The virtual layer uses 3D modeling, finite element simulation, and dynamic reduction technology to create a virtual model based on the physical layer’s actual properties and data. This model mirrors the physical layer in a virtual environment. This layer comprises geometric, finite element simulation, and a dynamic model reduction. These three types of models work together to construct an accurate virtual DT model in the virtual layer space, capable of mapping the geometric shape, physical properties, behavioral rules, and operational status of the gear pair.

The service layer refers to the application of the DT model, which can provide practical, multifunctional services to real-world application domains through the redevelopment of model functionality. Within this framework, it primarily offers parameter optimization services based on the WHO and reliability assessment services based on the WPHM. By collecting interaction data from the physical layer and virtual layer, extracting feature information from vibration acceleration data, and employing data-driven and model-driven methods, vibration acceleration is converted into parameter optimization results and reliability assessment results.

### 2.2. Dynamic Model Reduction Theory

Model reduction is a class of methods that maps the state space of high-dimensional systems onto low-dimensional subspaces via mathematical projection. The aim is to construct computationally efficient models that preserve the original system’s key characteristics. Dynamic model reduction is a significant approach within this category that focuses on retaining the dynamic features of the system’s state evolution over time. This approach constructs reduced-order models that can describe transient processes. This paper uses the Proper Orthogonal Decomposition (POD) method to create a dynamic reduced-order model (ROM) of a gear pair in a high-speed train gearbox.

#### 2.2.1. ROM Theory

The ROM generates transient simulation results using torque excitation inputs. A scenario is defined as the encapsulation of both the torque excitations history B(t) and the corresponding temporal output vectors X(t). The goal of the learning phase is to identify from a set of learning scenarios an optimal nonlinear function f that approximates the derivative $X^{\prime }(t)$ such that:

(1) $$\begin{array}{c} X^{\prime }(t)=f\left(X(t),B(t)\right)\\ X(t=0)=X_{0} \end{array}$$

where $X^{\prime }(t)$ is the time derivative of X(t), f is a nonlinear function of X(t) and B(t), $X_{0}$ is a vector of size n. It is the initial condition, that is, the solution at the first timestep.

f is determined by an optimization process aiming at minimizing $∥X_{ROM}(t)-X(t){∥}^{2}$. The originality of the process lies in the addition of free variables to account for higher nonlinearities, time lags, or hysteresis. It is defined as an iterative process which consists of progressively adding I(t) variables in Equation (2) until the precision required is reached:

(2) $$\left(\begin{array}{c} X^{\prime }(t)\\ I^{\prime }(t) \end{array}\right)=f\left(\left(\begin{array}{c} X(t)\\ I(t) \end{array}\right),G(t)\right)$$

#### 2.2.2. POD Theory

The core principle of proper orthogonal decomposition is singular value decomposition. Let M(t) be a set of dynamic signals (n time dependent signals with $n_{t}$ time steps). With Singular Value Decomposition (SVD), the original set of signals can be represented as follows:

(3) $$M=U\Sigma V^{*}$$

where U is a unitary matrix, Σ is a diagonal, and $V^{*}$ is a unitary matrix.

SVD lets you approximate the matrix M in a basis of r modes. Thus, the matrix M can be approximated by $M_{red}$, its projection in the r first left singular vectors basis:

(4) $$M≜M_{red}=U_{r}\cdot \Sigma_{r}\cdot V_{r}^{*}=U_{r}\cdot C$$

where $U_{r}$ is the projection basis, C is the dynamic modes coefficients that will be used as inputs or outputs of the dynamic ROM.

The dynamic reduction in the high-dimensional full-order model is now complete.

## 3. Reliability Assessment Modeling via WHO-Optimized WPHM

### 3.1. WHO Optimization Algorithm

**Figure 3:**
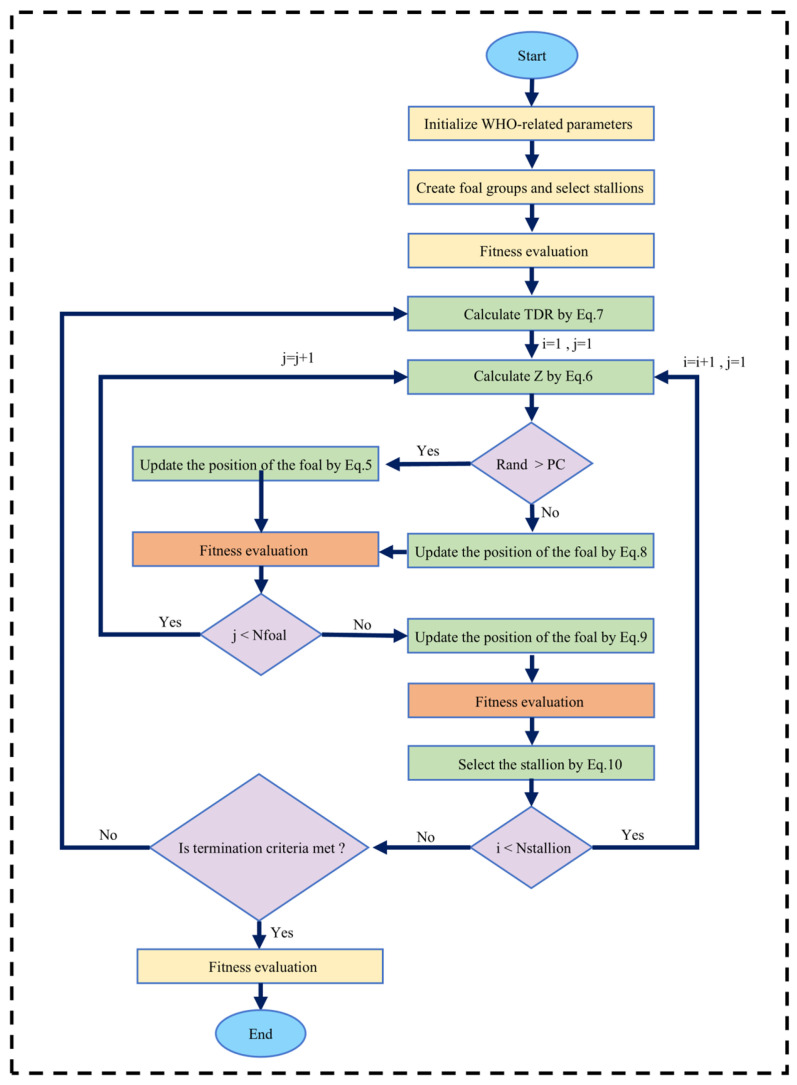
Flowchart of the WHO.

WHO is a novel swarm intelligence algorithm derived from the behavioral patterns observed in wild horse populations, including grazing, chasing, dominance, leadership, and mating. This algorithm exhibits strong evolutionary capabilities, rapid convergence, and robust optimization performance [24]. Figure 3 illustrates the flowchart of the WHO, which comprises five primary stages.

#### 3.1.1. Population Initialization

First, we divide this initial population into several groups. If N is the number of members of the population, the number of groups is G=N×PS. The PS is the percentage of stallions in the total population that we consider as a control parameter for the proposed algorithm. So, we have the leader G (stallion) according to the number of groups, and the remaining members (N−G) are divided equally among these groups.

#### 3.1.2. Grazing Behavior

Young horses predominantly occupy their time grazing within their group’s vicinity. To simulate this grazing behavior, the stallion is designated as the centroid of the grazing area, with group members performing localized search around this central point. During this simulated grazing, members explore around the leader stallion within variable search radii. Their positional update is mathematically defined as follows:

(5) $${\bar{X}}_{i,G}^{j}=2Z\cos(2\pi RZ)\times \left({\mathrm{Stallion}}^{j}-X_{i,G}^{j}\right)+{\mathrm{Stallion}}^{j}$$

where ${\bar{X}}_{i,G}^{j}$ denotes the current position vector of a group member (foal or mare), Stallion the position vector of the leader, and R a uniformly distributed random variable [−2, 2]. The term R governs grazing directionality by generating angular displacement (360°) relative to the leader. Combining the COS function with R causes movement at different radii, mainly controlling the angle between individuals and leaders.

The adaptive mechanism Z is computed as follows:

(6) $$P=R_{1}<TDR;\;\;\;IDX=(P==0);\;\;\;Z=R_{2}\Theta IDX+R_{3}\Theta (∼IDX)$$

where P is a vector consisting of 0 and 1 equal to the dimensions of the problem, Θ is a dot product, $R_{1}$ and $R_{3}$ are random vectors with uniform distribution in the range [0, 1], $R_{2}$ is a random number with uniform distribution in the range [0, 1], IDX indexes of the random vector $R_{1}$ returns that satisfy the condition (*P* == 0), TDR is the coefficient that linearly decreases from 1 to 0.

(7) $$TDR=1-iter\frac{1}{\max iter}$$

where iter is the current iteration, and maxiter is the maximum number of iterations of the algorithm.

#### 3.1.3. Mating Behavior

One of the unique behaviors of horses, compared to other animals, is to separate foals from the herd and mate with them. Foals vacate their familial groups prior to puberty onset, with males typically integrating into bachelor herds and females transferring to unrelated family units. This dispersal strategy mitigates inbreeding by preventing copulation between sires and their filial offspring or among siblings.

To simulate the behavior of the departure and mating of horses, which is the same as the Crossover operator of the mean type, has been proposed.

(8) $$X_{G,K}^{p}=Crossover(X_{G,i}^{q},X_{G,j}^{z}),i\neq j\neq k,p=q=end,Crossover=Mean$$

where $X_{G,K}^{p}$ is the position of the horse p from the group k, and leave the group and gives its place to a horse whose parents are horses who have to leave the group i and j have reached puberty. $Crossover(X_{G,i}^{q},X_{G,j}^{z})$ dispersal of foal q from group i and its subsequent breeding with foal z (from distinct group j) upon attaining reproductive maturity.

#### 3.1.4. Population Leadership

In natural ecosystems, population leaders primarily guide their groups to optimal habitats. When a habitat becomes dominated by a rival population, the displaced group is compelled to relocate. The next position of the leader relative to the habitat in each population is as follows:

(9) $$\bar{Stallion_{G_{i}}}=\left\{\begin{array}{c} 2Z\cos(2\pi RZ)\times (WH-Stallion_{G_{i}})+WH,\mathrm{if}\;R_{3}>0.5\\ 2Z\cos(2\pi RZ)\times (WH-Stallion_{G_{i}})-WH,\mathrm{if}\;R_{3}\leq 0.5 \end{array}\right.$$

where $\bar{Stallion_{G_{i}}}$ is the next position of the leader of the i group, WH is the position of the water hole, $Stallion_{G_{i}}$ is the current position of the leader of the i group, R is a uniform random number in the range [−2, 2].

#### 3.1.5. Exchange and Selection of Leaders

Initial leader selection employs stochastic sampling to preserve algorithmic randomness. During later iterations, selecting leaders based on fitness. When a group member demonstrates superior fitness to the current leader, their positions undergo a competitive exchange according to:

(10) $$Stallion_{G_{i}}=\left\{\begin{array}{c} X_{G_{i}},\mathrm{if}\;cost(X_{G_{i}})<cost(Stallion_{G_{i}})\\ Stallion_{G_{i}},\mathrm{if}\;cost(X_{G_{i}})>cost(Stallion_{G_{i}}) \end{array}\right.$$

The maximization objective function is the center position in the horse herd.

The WHO parameter optimization process is completed through the above steps.

### 3.2. LTSA Algorithm

**Figure 4:**
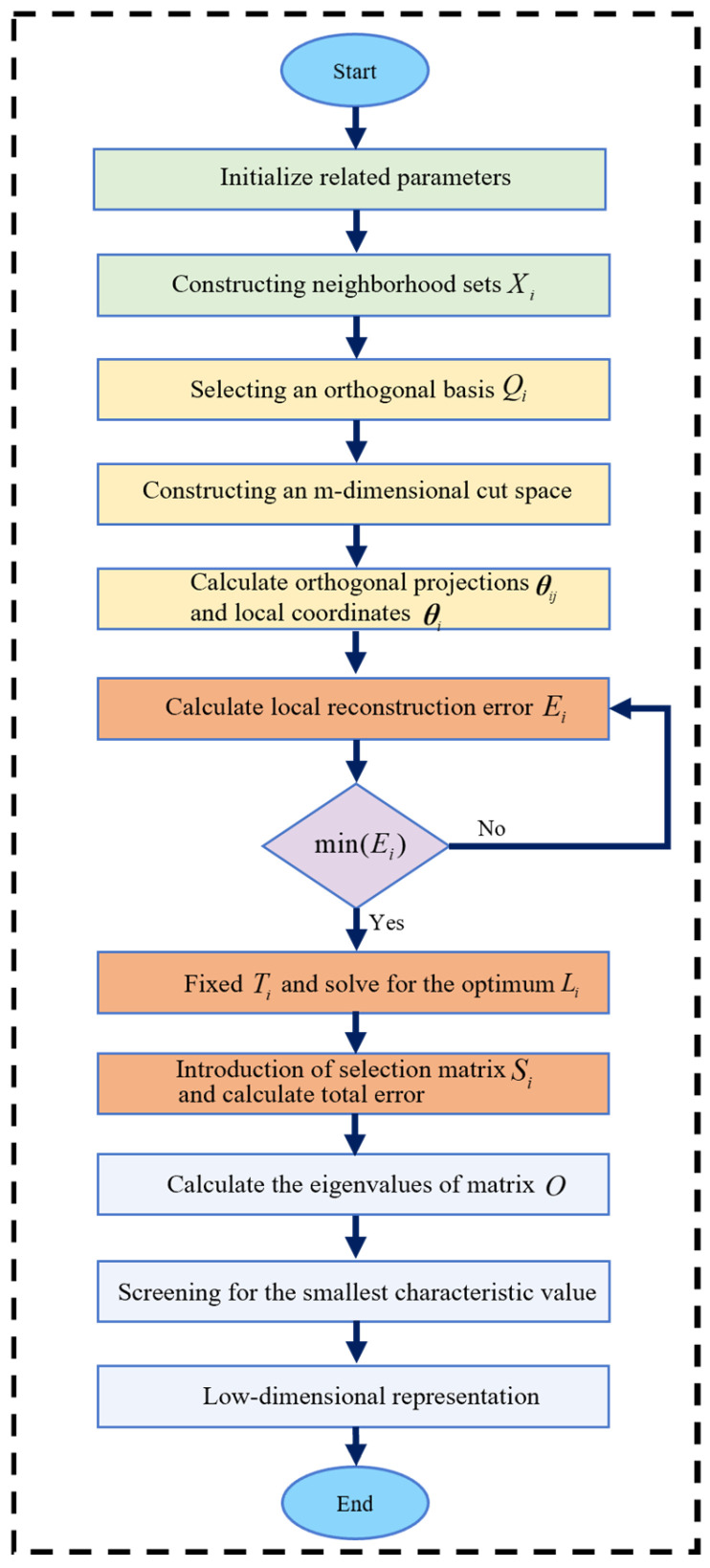
LTSA algorithm flowchart.

Vibration acceleration signals capture the gearbox’s operational status through both time-domain and frequency-domain characteristics. However, excessive features can induce redundancy, compromising the efficiency and accuracy of reliability assessment. Consequently, high-dimensional feature vectors require dimensionality reduction, achieved herein using the LTSA algorithm. Let $X=\{x_{1},x_{2},\ldots ,x_{N}\}$ denote a high-dimensional feature sample set in space $R^{a}$, with the specific workflow depicted in Figure 4.

#### 3.2.1. Extraction of Local Neighborhood Characteristics

For any point $x_{i}$ in the high-dimensional sample space $R^{m}$, determine the k nearest points with the smallest Euclidean distances containing $x_{i}$, and generate the neighborhood $X_{i}=\{x_{i1},x_{i2},\ldots ,x_{ik}\}$ of $x_{i}$.

#### 3.2.2. Local Linear Fitting

Select an orthogonal basis $Q_{i}$ from the vectors in $X_{i}=\{x_{i1},x_{i2},\ldots ,x_{ik}\}$ to construct the m-dimensional tangent space of $x_{i}$. Calculate the orthogonal projection $\theta_{ij}=Q_{i}^{T}(x_{ij}-{\bar{x}}_{i})$ of each point in $X_{i}=\{x_{i1},x_{i2},\ldots ,x_{ik}\}$ onto the tangent space. Describe the low-dimensional set structure of point $x_{i}$ through the local coordinates of orthogonal projection:

(11) $$\left\{\theta_{i1},\theta_{i2},\ldots ,\theta_{ik}\}=\{Q_{i}^{T}(x_{i1}-{\bar{x}}_{i}),Q_{i}^{T}(x_{i2}-{\bar{x}}_{i}),\ldots ,Q_{i}^{T}(x_{ik}-{\bar{x}}_{i})\right\}$$

where ${\bar{x}}_{i}$ is the mean value of $x_{i}$.

#### 3.2.3. Local Coordinate Global Arrangement

Let $L_{i}$ denote the local coordinate system and $T_{i}=\{t_{i1},t_{i1},\ldots ,t_{ik}\}$ represent the global coordinate system. At this location, the local reconstruction error is as follows:

(12) $$E_{i}=T_{i}(I-\frac{1}{k}ee^{T})-L_{i}\theta_{i}$$

where I denote the identity matrix and e the all-ones vector.

Optimize $T_{i}$ and $L_{i}$ by minimizing the sum of reconstruction errors for all sample points, thereby ensuring the integrity of data information embedded in low-dimensional space.

(13) $$\min\sum_{i=1}^{N}{E_{i}}^{2}=\min\sum_{i=1}^{N}T_{i}(I-\frac{1}{k}ee^{T})-L_{i}{\theta_{i}}^{2}$$

Fix $T_{i}$ to obtain the optimal permutation matrix $L_{i}$.

(14) $$L_{i}=T_{i}(I-\frac{1}{k}ee^{T})\theta_{i}^{+}$$

where $\theta_{i}^{+}$ is the Moore–Penrose generalized inverse of $\theta_{i}$.

The local reconstruction error $E_{i}$ is then given by:

(15) $$E_{i}=T_{i}(I-\frac{1}{k}ee^{T})(I-\theta_{i}^{+}\theta_{i})$$

Let $W_{i}=(I-\frac{1}{k}ee^{T})(I-\theta_{i}^{+}\theta_{i})$, $T=\{T_{1},T_{2},\ldots ,T_{N}\}$, $TT^{T}=I$, $S_{i}$ is a binary selection matrix, then $T_{i}=TS_{i}$, so the sum of the local reconstruction differences in all sample points is:

(16) $$\sum_{i=1}^{N}||E_{i}|{|}^{2}=\overset{N}{\underset{i=1}{\sum ||}}T_{i}(I-\frac{1}{k}ee^{T})(I-\theta_{i}^{+}\theta_{i})|{|}^{2}=\sum_{i=1}^{N}||TS_{i}W_{i}|{|}^{2}=||TSW|{|}_{\begin{array}{l} F \end{array}}^{2}$$

where $S=\{S_{1},S_{2},\ldots ,S_{N}\}$, $W=diag\{W_{1},W_{2},\ldots ,W_{N}\}$, $||\cdot |{|}_{F}^{2}$ represents the square of the Frobenius norm.

#### 3.2.4. Determine the Low-Dimensional Global Coordinate Mapping

Let $O=SWW^{T}S^{T}$, compute the eigenvalues of matrix O, The n+1 eigenvectors corresponding to the smallest eigenvalues are obtained. After discarding those associated with zero eigenvalues, the remaining n-dimensional eigenvectors form the low-dimensional embedding coordinates for the high-dimensional data.

### 3.3. Estimation of WPHM Parameters

The WPHM, which employs the Weibull distribution as the baseline hazard function, can be expressed as:

(17) $$h\left(t,Z\right)=\frac{\beta }{\eta }{\left(\frac{t}{\eta }\right)}^{\beta -1}\exp(\alpha Z)$$

where t represents the running time of the high-speed train, β denotes the shape parameter, η is the scale parameter, α corresponds to the covariate regression coefficient, and Z indicates the value of the state feature index at time t. The parameter set to be estimated is denoted by θ=(β,η,α). Given the observed time data $\left(t_{1},t_{2},\ldots ,t_{n}\right)$, the likelihood function can be derived as follows:

(18) $$L(\theta )=\prod_{i=1}^{D}h(t_{i},\theta )\prod_{j=1}^{N}R(t_{j},\theta )$$

where $R(t_{j},\theta )$ denotes the reliability function, $h(t_{i},\theta )$ represents the failure rate function, D corresponds to the failure set, and N indicates the sample set. By taking the logarithm of both sides of the likelihood function, the log-likelihood function is obtained as follows:

(19) $$\ln L(\theta )=r\ln(\frac{\beta }{\eta })+\sum_{i=1}^{r}\left[\left(\beta -1)\ln(\frac{t_{i}}{\eta })+\alpha Z(t_{i}\right)\right]-\sum_{j=1}^{n}{\left(\frac{t_{j}}{\eta }\right)}^{\beta }\exp[\alpha Z(t_{j})]$$

where n denotes the total data amount, r is the number of failures within n, $Z(t_{i})$ is the response covariate recorded at time $t_{i}$, and $Z(t_{j})$ is the response covariate at time $t_{j}$.

According to the principle of maximum likelihood estimation, setting the partial derivatives of lnL(θ) with respect to the parameters β, η, and α to zero yields a system of nonlinear equations:

(20) $$\left\{\begin{array}{c} \frac{\partial \ln L}{\partial \beta }=\frac{r}{\beta }+\sum_{i=1}^{r}\ln\left(\frac{t_{i}}{\eta }\right)-\sum_{j=1}^{n}{\left(\frac{t_{j}}{\eta }\right)}^{\beta }\ln\left(\frac{t_{j}}{\eta }\right)\exp\left[\alpha Z(t_{j})\right]\\ \frac{\partial \ln L}{\partial \eta }=-\frac{r\beta }{\eta }+\sum_{j=1}^{n}\frac{\beta }{\eta }{\left(\frac{t_{j}}{\eta }\right)}^{\beta }\exp\left[\alpha Z(t_{j})\right]\\ \frac{\partial \ln L}{\partial \alpha }=\sum_{i=1}^{r}Z(t_{i})-\sum_{j=1}^{n}{\left(\frac{t_{j}}{\eta }\right)}^{\beta }Z(t_{j})\exp\left[\alpha Z(t_{j})\right] \end{array}\right.$$

The system of equations given in Equation (20) was solved using the WHO. The fitness function was defined as the sum of the absolute values of all function outputs. Optimization was carried out by minimizing this fitness function. Upon stable convergence of the WHO, the solution to the system of equations was obtained, yielding the estimated parameters β, η, and α, thereby completing the parameter estimation for the WPHM.

## 4. Construction of a DT Model for a High-Speed Train Gearbox

**Figure 5:**
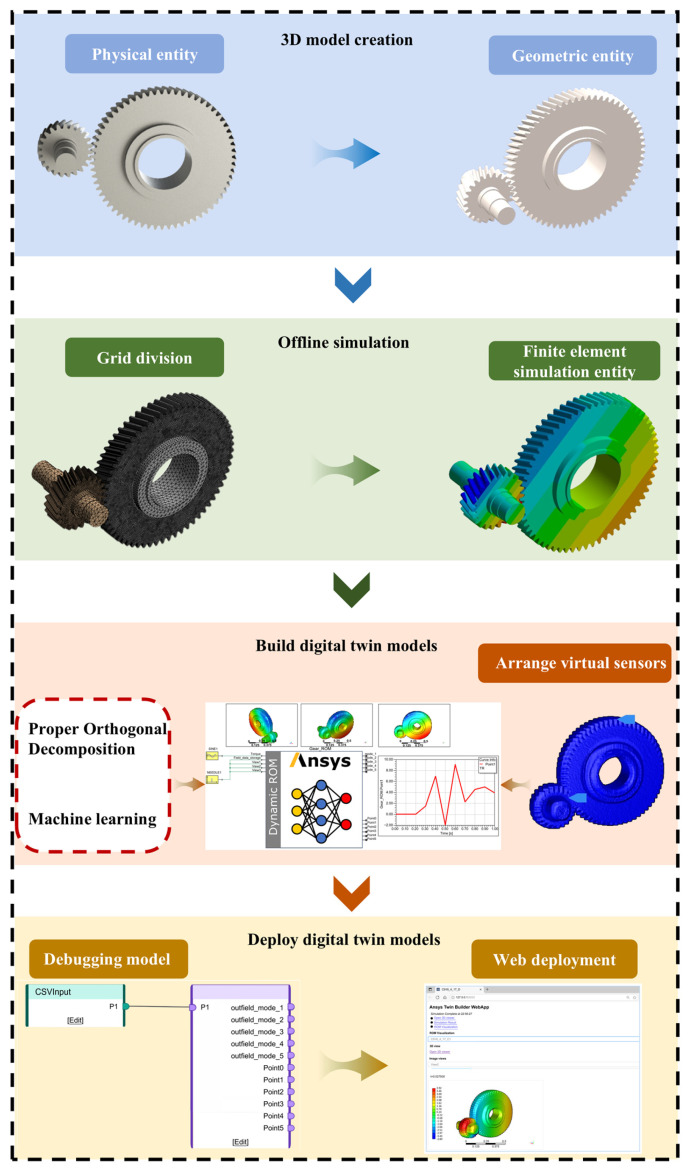
A technical roadmap for developing a DT model of a high-speed train gearbox.

This section uses a gear pair in a high-speed train gearbox as an example to create and deploy a DT model of the high-speed train gearbox. The creation process is divided into four modules: the 3D model creation module, the offline simulation module, the DT model construction module, and the DT model deployment module. The technical roadmap for creation and deployment is shown in Figure 5.

### 4.1. Geometric Modeling

**Figure 6:**
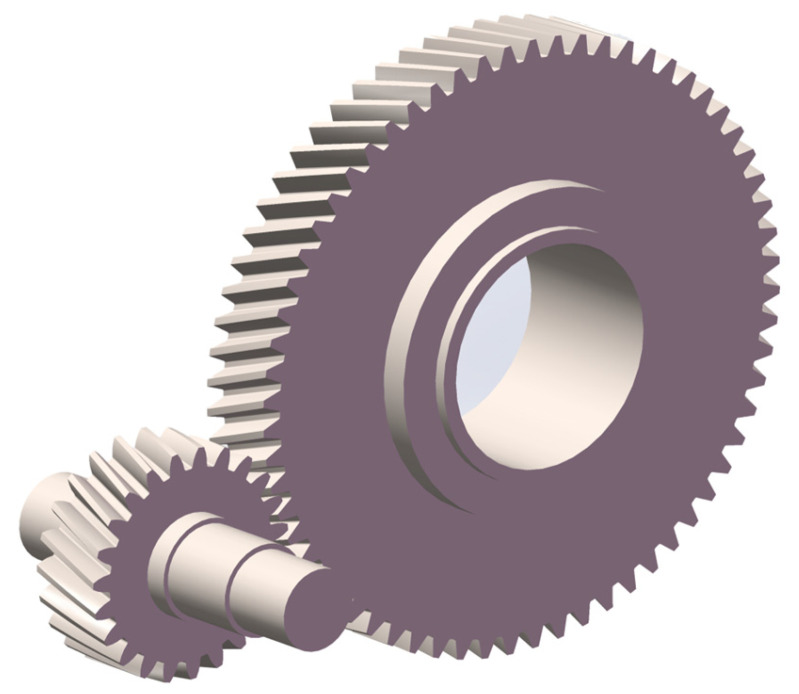
Three-dimensional model of gear pair in high-speed train gearbox.

Three-dimensional geometric model construction serves as the foundation for building the DT model. The establishment of a high-fidelity 3D geometric model enables accurate mapping of the structural parameters, dimensional properties, and assembly relationships of a high-speed train gearbox into virtual space. This allows the DT to achieve a “mirror-image” representation at the geometric level. The 3D model developed using SolidWorks is presented in Figure 6. All gears are involute gears. To balance computational efficiency and accuracy, no tooth profile modification or gear optimization was applied to the gears in this study. The structural parameters and geometric dimensions of the gears are listed in Table 1.

### 4.2. Offline Finite Element Simulation Analysis

Offline finite element simulation is an indispensable component in creating the DT model. Through transient dynamic simulation, it enables precise analysis of gear pair dynamic characteristics, mapping the physical properties and behavioral rules of the gear pair DT model in the virtual space. This process also captures the vibration acceleration response of the gear pair under conditions closely approximating real operating conditions.

**Figure 7:**
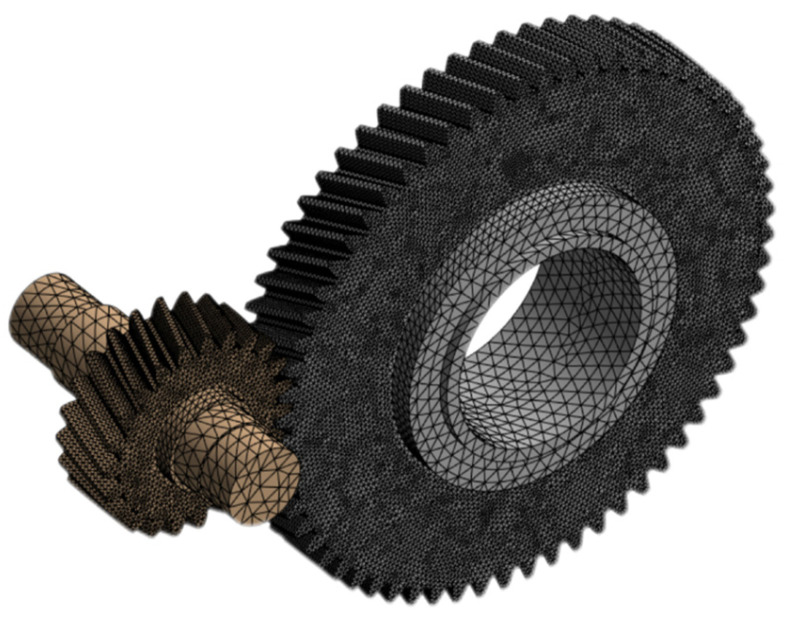
Finite element mesh generation for gear.

**Figure 8:**
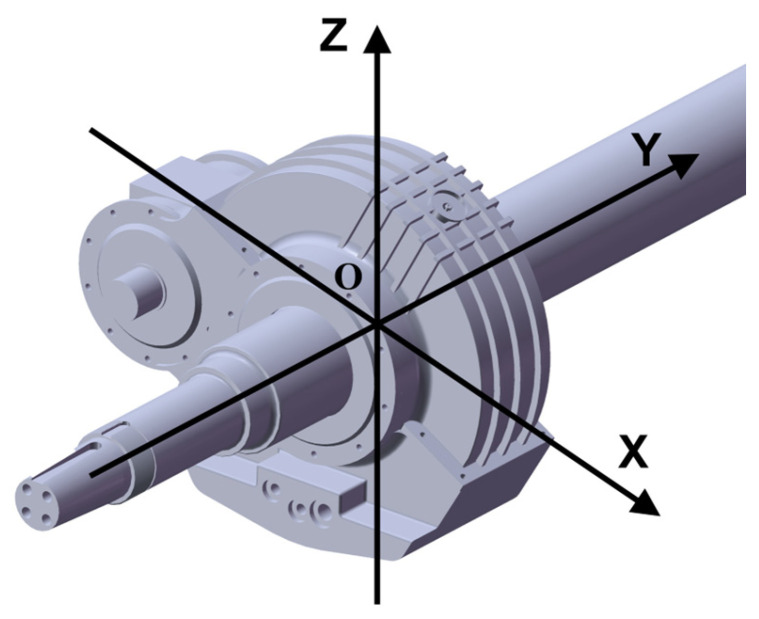
Direction of vibration signal.

In this study, finite element analysis was performed using Ansys Workbench. Under the operating conditions of a certain high-speed train traveling at 300 km/h, constraints were applied. A drive torque of 1255.71 N·m was added to the drive gear. A contact pair with a friction coefficient of 0.15 was added to the contact surfaces of the two gears. The material is 20CrMnTi. To balance computational resources and accuracy, a 15 mm tetrahedral mesh was used overall, with a 5 mm tetrahedral mesh applied to the meshing contact surfaces and gear flanks. Figure 7 shows the mesh partitioning diagram, with a total of 214,627 mesh elements and 362,247 nodes, achieving an average quality factor of 0.717. Higher solver accuracy requires fewer simulation steps for more precise results. The solver type was set to program-controlled with a minimum step size of 20, and the simulation duration was 1 s. This study acquired dynamic vibration responses and utilized vibration acceleration as input data for subsequent reliability assessment models. Under identical operating conditions, horizontal vibration signals typically provided more useful information for tracking degradation processes than vertical vibration signals. Therefore, the offline simulation analysis in this paper only required exporting the Y-direction vibration acceleration contour plot. The vibration signal direction was shown in Figure 8. The simulation results for vibration acceleration in the Y-direction have a range of −7.970 to 8.020 m/s^2^. This is within the engineering tolerance range of 5–10% deviation from the actual amplitude range of the data, which is −8.740 to 8.999 m/s^2^.

### 4.3. DT Model Construction

#### 4.3.1. Generate the ROM

This study uses the POD to create ROMs for a full-order finite element model. This process requires offline finite element simulation results and their corresponding spatial coordinate points. The underlying principle of the model order reduction is described in Section 2.2. Using the Dynamic ROM Builder module, the excitation and simulation results from Scenario_1 were selected in the Reduce Outputs settings. The target relative accuracy was set to 0.1%, and the maximum number of modes was limited to 20 for training the ROM. The resulting dynamic ROM substantially reduces computational time: the simulation time decreased from 82.4 h to 12.6 s, corresponding to a speed-up factor of 2.35 × 10^4^, thereby satisfying the real-time requirements of DT technology.

#### 4.3.2. Determine the Critical Measurement Points

**Figure 9:**
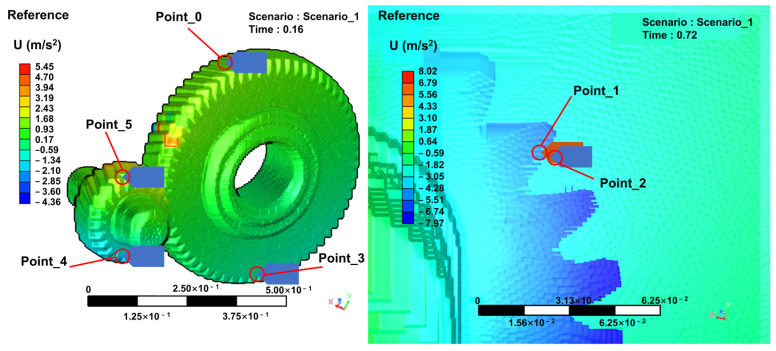
Virtual sensor placement in the DT model.

To overcome the limitation of physical sensor placement inside a high-speed train gearbox. This study optimizes the measurement point locations in the ROM and precisely deploys virtual sensors to improve the accuracy of reliable assessments from the data source level. Meshing impacts represent a significant source of excitation in gear transmission systems. In actual gear meshing, due to manufacturing and installation errors, as well as gear deformation, the meshing point deviates from the theoretical meshing point of the meshing line. When the rotational speed increases abruptly, the meshing force changes rapidly, leading to a transient meshing impact [25]. Owing to differences in geometric profile and mechanical strength between the drive and driven gear, two key measurement points are established at their meshing interface to enable simultaneous reliability evaluation of both components. As illustrated in Figure 9, virtual sensors are deployed at three locations: Point_0, Point_3, Point_4, and Point_5 are used for similarity validation against conventional sensor data, Point_1 outputs the vibration acceleration at the drive gear meshing point, and Point_2 outputs the vibration acceleration at the driven gear meshing point.

#### 4.3.3. Compiling and Packaging the DT Model

**Figure 10:**
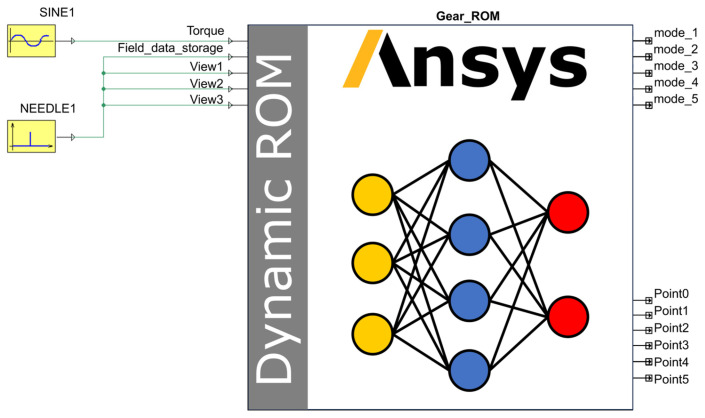
Dynamic ROM.

During the compilation and encapsulation of the DT model, the frequency control components SINE and NEEDLE were connected within the Ansys Twin Builder main interface to regulate the input frequency of the ROM. The resulting dynamic ROM is illustrated in Figure 10. Where the input port “Torque” represents the gear torque input, while “Field_data_storage” is utilized to manage modal coefficients and write snapshot files. Components “View1”, “View2”, and “View3” control the frame rate of the visualization contour plot animation. Outputs “mode_1” to “mode_5” represent these five output modes. Additionally, “Point0”, “Point1”, “Point2”, “Point3” “Point4”and “Point5” provide virtual sensor data corresponding to the locations Point_0, Point_1, Point_2, Point_3, Point_4 and Point_5, defined in Figure 10, respectively.

**Figure 11:**
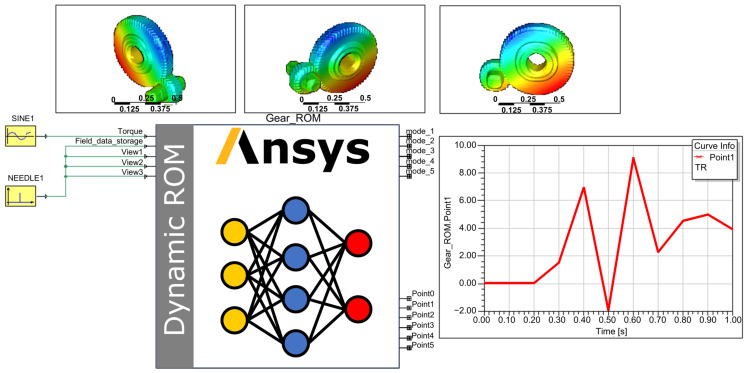
DT system for gear transmission.

**Figure 12:**
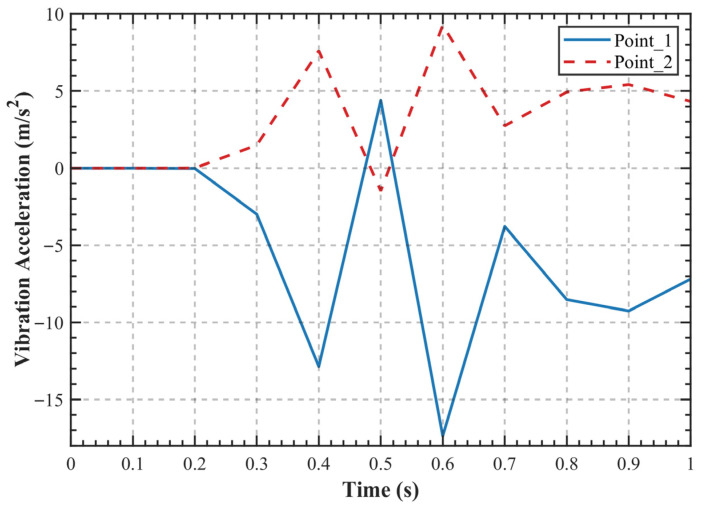
Virtual sensor data.

Within the Ansys Twin Builder environment, the components of the control ROM were interconnected to form a complete DT system, as illustrated in Figure 11. Where SINE1 regulates the torque input to the ROM, while NEEDLE1 controls the modal coefficients, manages the writing of snapshot files, and adjusts the frame rate of the visualization contour plot animation. Above the ROM, visualization windows provide three distinct viewing perspectives of the gear pair, enabling direct observation of real-time contour plot changes during simulation. The chart located to the right of the ROM allows real-time monitoring of the dynamic response curve from virtual sensor Point_1. The output curves of virtual sensors Point_1 and Point_2 are further presented in Figure 12.

**Figure 13:**
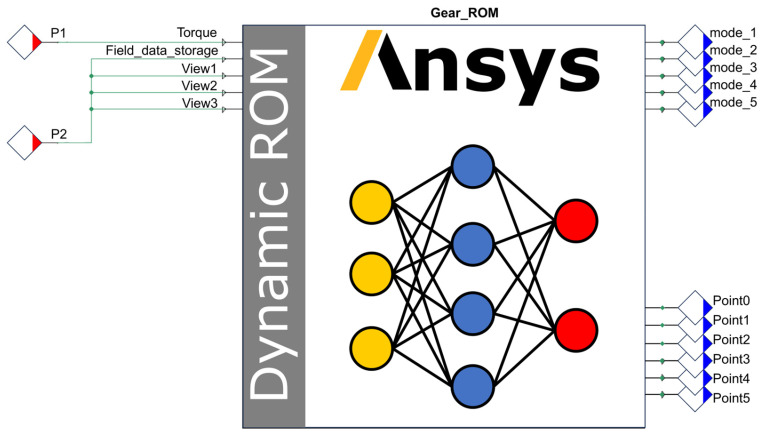
Encapsulating the DT model.

Model encapsulation entails defining the input and output interfaces of the gear pair DT system. The SINE1 module is mapped to interface P1, while the NEEDLE1 module is mapped to interface P2. Using the built-in encapsulation functionality of the software, the DT system is compiled into a standalone twin file, thereby enabling its deployment in the subsequent phase. The encapsulated DT system is illustrated in Figure 13.

### 4.4. DT Model Deployment

In the previous sections, a DT system for a high-speed train gear pair has been constructed, incorporating capabilities such as virtual–physical mapping, visualization, and real-time simulation. However, the current implementation relies on historical data. To incorporate real-time data from physical systems, the DT model must be deployed in an actual operational environment using Ansys Twin Deployer. The Ansys Twin Deployer 2022 R1 supports four deployment methods: Python App Generation, PTC Agent Generation, Twin WebApp Generation, and DTDL Generation. This study uses the Twin WebApp Generation approach to produce a software development kit (SDK) package that can be easily and rapidly deployed and executed via a web browser. The generated SDK package enables connection to live field data and supports DT simulations using real-time input.

**Figure 14:**
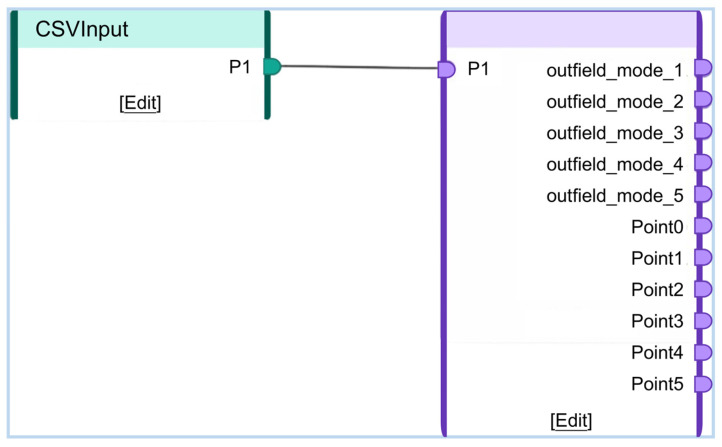
Ansys Twin Deployer deployment interface.

**Figure 15:**
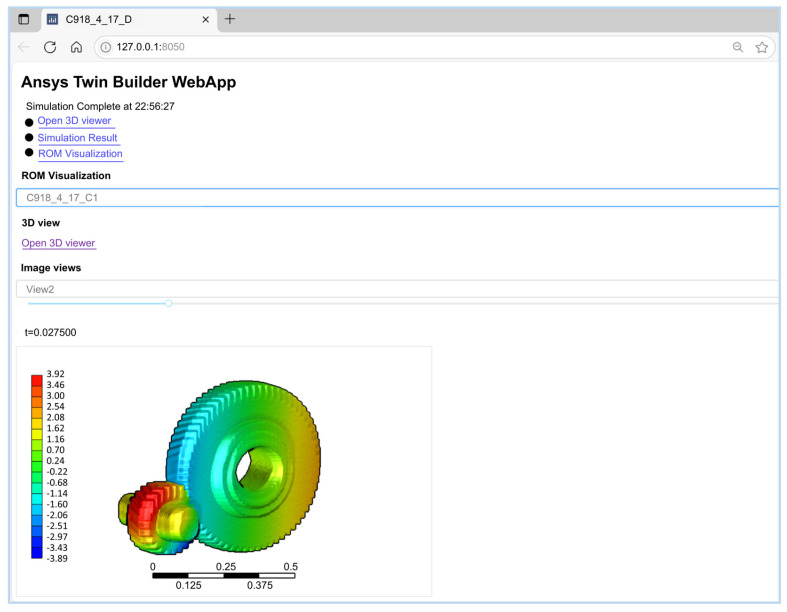
Web Deployment of a DT for a High-Speed Train Gear Pair.

Figure 14 illustrates the deployment interface within the Twin Deployer environment. Historical field data collected by sensors is imported into the CSVInput module, while the compiled and packaged twin file is loaded into the TwinModel component, with interface connections established at P1. Following pre-deployment data debugging, the twin model is ultimately deployed to a local web server via the generated SDK package, as shown in Figure 15.

### 4.5. DT Data Verification

**Figure 16:**
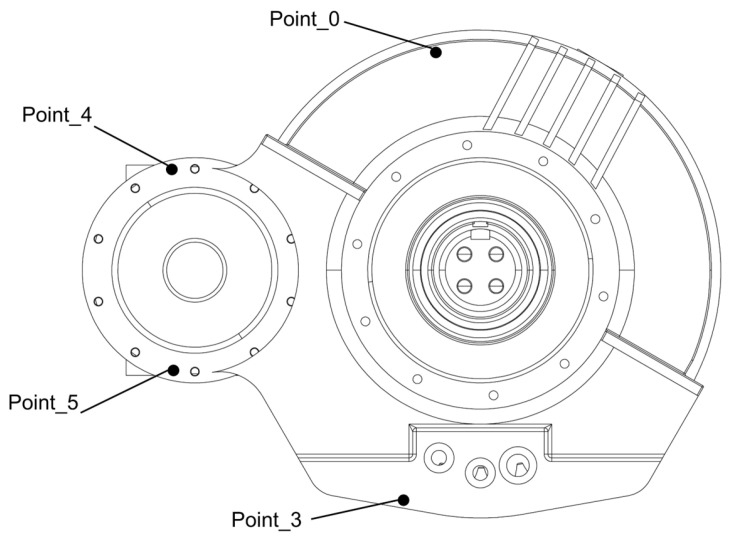
Sensor Layout for Actual Measurement Data.

Vibration is an inherent property of a high-speed train gearbox. This study evaluated the usability of the DT data by comparing the cosine correlation, root mean square error, and maximum cross-correlation coefficient between the virtual sensor signals of the DT and the time-domain response signals of physical sensors under actual operating conditions. Due to structural constraints of the high-speed train gearbox, most physical sensors are currently mounted on the gearbox housing. The sensor layout based on actual measurement data is shown in Figure 16. The verification comparison between DT signals and actual operational data at measurement Point_0, Point_3, Point_4, and Point_5 is shown in Table 2.

As shown in Table 2, this study evaluated the usability of the DT data across three dimensions: the shape, amplitude, and phase of the vibration acceleration signals. The results showed that in terms of cosine similarity, all measurement points yielded values greater than 0.6, with Point_0 having the lowest value (0.878) and Point_3 having the highest value (0.962). The RMSE at measurement point 0 was the only one to exceed 5%, at a value of 5.147%, whereas all other points remained below this threshold, thereby satisfying practical engineering requirements. For the cross-correlation coefficient, all points yielded values above 0.6; the minimum was 0.879 at point_0, and the maximum was 0.963 at point_3.

**Figure 17:**
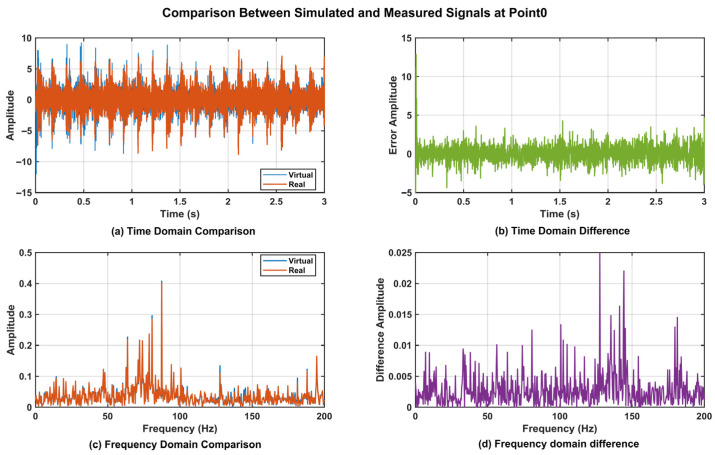
Comparison of DT Signal and Actual Signal at Point_0.

**Figure 18:**
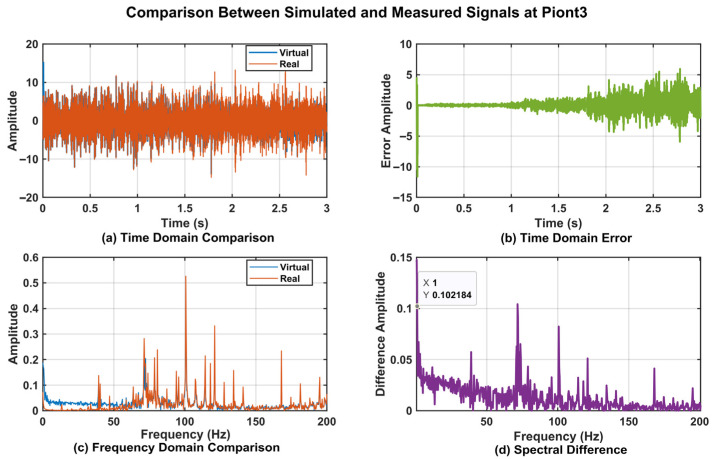
Comparison of DT Signal and Actual Signal at Point_3.

**Figure 19:**
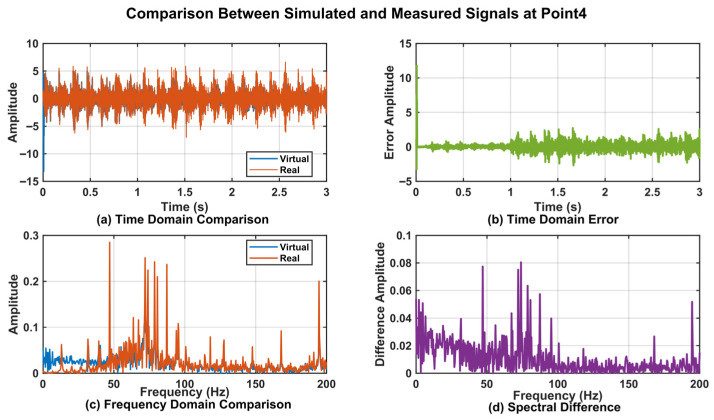
Comparison of DT Signal and Actual Signal at Point_4.

**Figure 20:**
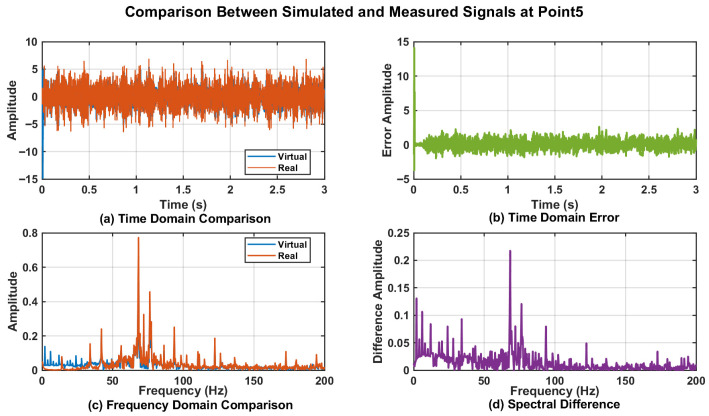
Comparison of DT Signal and Actual Signal at Point_5.

To further validate the effectiveness of the proposed DT data, a time-domain analysis was conducted on the vibration data from Point_0, Point_3, Point_4, and Point_5. The corresponding time-domain responses are presented in Figure 17a, Figure 18a, Figure 19a, and Figure 20a. The results demonstrated that the digital twin signals and the measured signals exhibited strong consistency in both amplitude variation trends and periodic characteristics. Regarding time-domain errors, Figure 17b showed that, except at 0.007 s and 0.011 s, where the errors exceeded 5 m/s^2^, the errors at all other time points remained within the range of [−5, 5 m/s^2^]. In Figure 18b, the errors exceeded 5 m/s^2^ during the initial phase (0.005 s, 0.007 s, 0.009 s, and 0.011 s). Although the errors increased slightly at later time points, the maximum error was only 5.931 m/s^2^. In Figure 19b, the errors exceeded 5 m/s^2^ only at 0.007 s and 0.010 s, while at all other time points they remained confined to the range of [−3, 3 m/s^2^]. At 0.005 s, 0.007 s, and 0.010 s in Figure 20b, the values exceeded 5 m/s^2^, while the error ranges at the remaining time points were similar to those of Point_4, also remaining within [−3, 3 m/s^2^]. Further analysis using Fast Fourier Transform (FFT) revealed the spectra for the four measurement points (Figure 17c, Figure 18c, Figure 19c, and Figure 20c), each showing distinct peak frequencies with largely consistent peak positions. Spectral error analysis (Figure 17d, Figure 18d, Figure 19d, and Figure 20d) indicated maximum errors of 0.025 m/s^2^, 0.150 m/s^2^, 0.080 m/s^2^, and 0.217 m/s^2^ for the respective measurement points.

In summary, this study verified the validity and usability of the DT data through comprehensive data and time-frequency domain analyses comparing the vibration acceleration signals with the measured signals. Since the DT points were positioned directly on the gear, they offered more direct and precise data acquisition locations compared to the physical sensors mounted on the housing. Although numerical discrepancies existed, both datasets demonstrated excellent consistency in key vibration characteristics, such as amplitude variation trends and periodic features.

## 5. Reliability Assessment of High-Speed Train Gearbox

**Figure 21:**
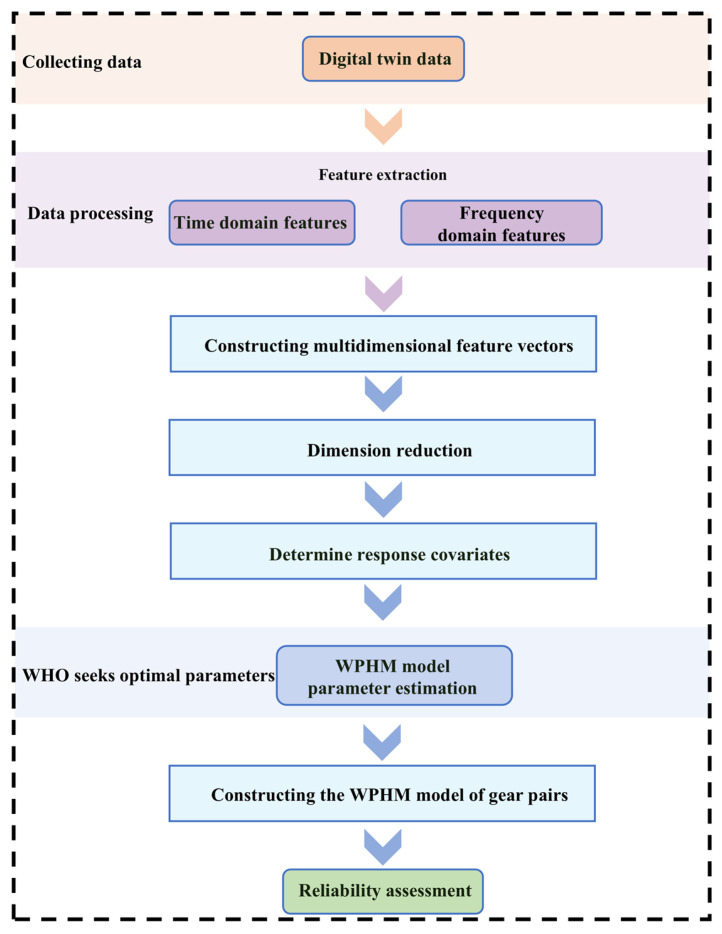
Flowchart for assessing the operational reliability of a high-speed train gearbox gear pair.

This study proposed a reliability assessment method that integrated the WHO and WPHM. Time-domain and frequency-domain feature parameters were extracted from vibration acceleration data to characterize performance degradation trends. A high-dimensional feature vector was then constructed for critical components of high-speed train gearboxes. The intrinsic dimension of the vibration data was determined using maximum likelihood estimation (MLE). The LTSA algorithm was then applied to reduce this vector. These reduced response covariates then served as inputs for the WPHM. The maximum likelihood function for the WPHM was then established, and optimal parameters were estimated using intelligent optimization algorithms. Finally, the complete WPHM was constructed using these optimal parameters to assess the reliability of critical components in high-speed train gearboxes. Figure 21 illustrated the reliability assessment workflow.

### 5.1. A Comparison of the Methods Used to Evaluate the Parameters of the WPHM Reliability Model

**Figure 22:**
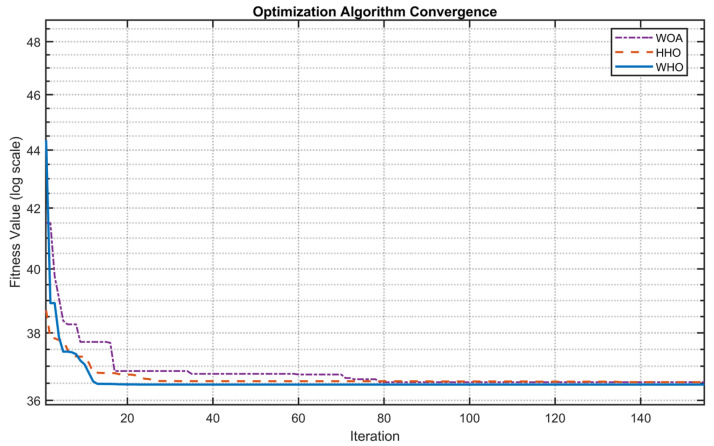
Convergence curves of the three algorithms for WPHM parameter optimization.

If the widely used maximum likelihood estimation algorithm is employed to solve for the parameters β, η, and α in Section 3.2, it may suffer from low computational efficiency and poor convergence. To analyze the performance of common optimization algorithms in solving the WPHM parameters, this study uses the WHO (Whale Optimization Algorithm), WOA (Whale Optimization Algorithm), and HHO (Harris Hawks Optimization) algorithms to optimize the WPHM parameters based on virtual measurement point data at Point_1. The parameter settings were referenced from existing applications of the WHO case [26]. To achieve a better global optimum solution, specific WHO parameters were configured, as summarized in Table 3. All three algorithms used the maximum number of function evaluations as the termination criterion, with a population size of 100 and a maximum of 300 iterations. Convergence behavior was monitored by tracking the best fitness value at each iteration, and the quality of the parameter estimation was evaluated based on these values. The fitness values for the three algorithms were shown in Table 4. As illustrated in Figure 22, the WHO exhibited a faster convergence rate compared to both WOA and HHO, attaining the global optimum in only 13 iterations. This represents a reduction in iteration count of 62.9% relative to WOA (35 iterations) and 48.1% compared to HHO (27 iterations). Furthermore, WHO achieved marginally superior solution quality, with fitness values 0.069% lower than those of WOA and 0.175% lower than HHO. These results confirm the accuracy and effectiveness of the WHO in conjunction with the WPHM for the reliability assessment of the high-speed train gearbox.

### 5.2. Reliability Assessment of Key Components in High-Speed Train Gearbox

Based on the results in Section 5.1, this study applied the WHO algorithm to solve for the optimal parameters to be estimated in the WPHM. As the DT model of the high-speed train gearbox described in this study is an ideal dynamic model that does not consider environmental or aerodynamic noise or other interference, the DT data does not require noise reduction processing. This study employs virtual measurement points, Point_1 and Point_2, in conjunction with the WHO, to estimate the parameters of the WPHM. The resulting parameter estimates are summarized in Table 5.

Use the parameters obtained from Table 5 to substitute into the reliability function and establish a complete WPHM reliability assessment model. The corresponding numerical expression is as follows:

(21) $$\left\{\begin{array}{l} R_{p}(t,Z)=\exp\left(-{\left(\frac{t}{42574.116}\right)}^{1.8370}\exp(0.502\cdot Z-0.170\cdot Z+0.247\cdot Z)\right)\\ R_{g}(t,Z)=\exp\left(-{\left(\frac{t}{44683.941}\right)}^{1.8370}\exp(0.589\cdot Z+1.105\cdot Z+0.918\cdot Z)\right) \end{array}\right.$$

where $R_{p}$ denotes the reliability of the drive gear, $R_{g}$ represents the reliability of the driven gear, and Z represents the low-dimensional response covariate derived from LTSA-based dimensionality reduction.

**Figure 23:**
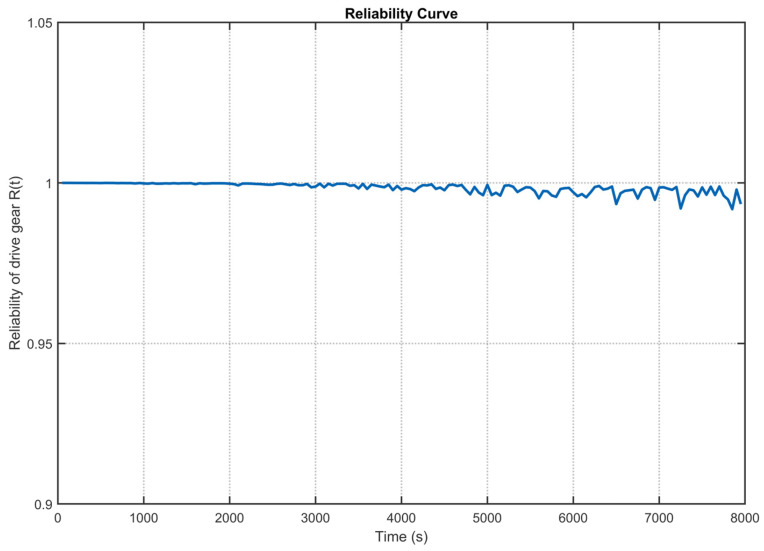
Reliability of the drive gear in a high-speed train gearbox.

**Figure 24:**
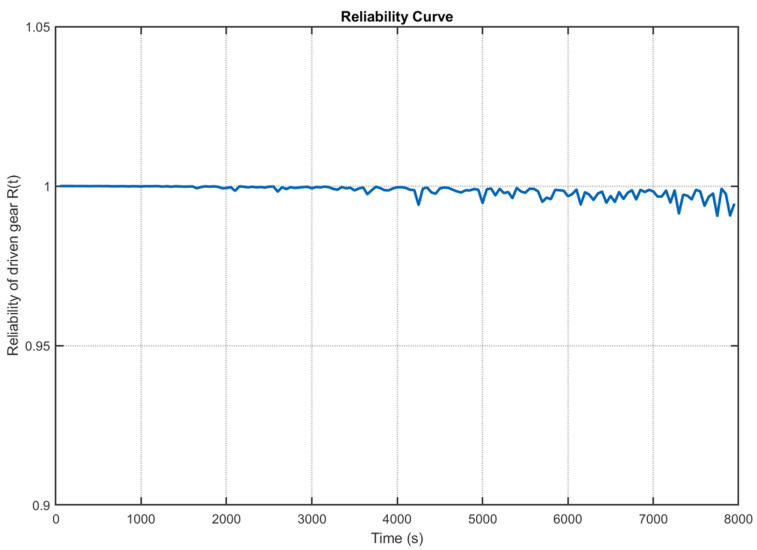
Reliability of the driven gear in a high-speed train gearbox.

Substituting the response covariates obtained from the DT data into Equation (21), the reliability of the drive gear is shown in Figure 23, and the reliability of the driven gear is shown in Figure 24. Figure 23 illustrates the reliability trend of the drive gear over time. Over the 0–8000 s interval, the reliability remains consistently high, approaching unity and indicating excellent operational stability. A slight, practically negligible decline is observed in the later phase (5000–8000 s), with the minimum reliability value recorded at 0.992. Similarly, Figure 24 depicts the reliability of the driven gear throughout the operating period. The reliability remains near 1.0 during the initial phase, reflecting a high degree of operational integrity. By the 8000 s mark, the reliability exhibits only minor fluctuations while largely maintaining stability, reaching a minimum value of 0.991. Within the operating range of the high-speed train, which is 0 to 8000 s, both the drive and driven gears demonstrate high reliability, with a reliability factor that consistently exceeds 0.991. They remain in Class I maintenance status, requiring only routine inspections, and meet the project’s practical requirements.

## 6. Conclusions

This study applies DT technology to the reliability assessment of a high-speed train gearbox, addressing a critical gap left by traditional physical sensors, which are unable to collect operational data from key internal components. Using a gear pair within the gearbox as an example, a digital replica incorporating realistic material properties and operating conditions was developed through simulation and modeling. An ROM of the gear pair was constructed via dynamic model reduction, achieving a simulation acceleration factor of 2.35 × 10^4^, thereby ensuring computational efficiency and real-time capability of the DT. Virtual sensors were deployed at the gear meshing points to collect vibration acceleration data, which was visualized in real time through line charts, enabling effective monitoring at critical internal locations. Furthermore, the Ansys Twin Deployer was employed to deploy the DT system on a web-based platform, integrating the model into practical engineering applications. Using DT data acquired from virtual measurement points as input, this study combines the WHO and the WPHM to achieve an accurate reliability assessment of the gear pair. This approach provides a valuable reference for predictive maintenance and operational decision-making regarding a high-speed train gearbox.

While this study focuses on a gear pair as a representative example to demonstrate the implementation of DT technology, future work will expand toward constructing a comprehensive DT of the entire gearbox system under various operating conditions. Subsequent research will further explore applications of DT in reliability analysis and enhance the functional capabilities of the twin model. Furthermore, the accuracy of the constructed DT model was validated using actual operational historical data from four measurement points. However, the model’s performance in real time under actual operating conditions has yet to be validated. Future research may deploy DT models in actual operating environments, such as bench tests or in-service high-speed train gearbox systems, to further validate the dynamic response performance of the models and the accuracy of DT data.

## Figures and Tables

**Table 1:** Gear Structural Parameters and Dimensional Parameters.

Parameters	Value	Parameters	Value	Parameter	Value
Number of teeth on the drive gear	23	Number of teeth on the driven gear	64	Tooth top coefficient	1
Normal displacement coefficient of the drive gear	0.45	Normal displacement coefficient of the driven gear	−0.20	Root coefficient	0.25
Modulus	8	Helix angle	23	Pressure angle	20
Tooth width	70 mm	Center distance	378 mm		

**Table 2:** Comparison of DT Signals and Actual Measurement Signals.

Measurement Point	Cosine Similarity	RMSE	Cross-Correlation Coefficient
Point_0	0.878	5.147%	0.879
Point_3	0.962	3.935%	0.963
Point_4	0.934	3.727%	0.935
Point_5	0.939	3.886%	0.940

**Table 3:** Parameters of the WHO.

Parameters	Value
Number of iterations	100
Population size	300
Crossover type	Mean
Crossover percentage	0.1
Stallions percentage	0.2

**Table 4:** Fitness values of the three optimization algorithms.

Algorithm Type	Fitness Value
WOA	36.484
HHO	36.523
WHO	36.459

**Table 5:** Parameter estimates for the WPHM.

WPHM Parameters	Drive Gear	Driven Gear
β	1.8370	1.8370
η	42,574.116	44,683.941
$\alpha_{1}$	0.502	0.589
$\alpha_{2}$	−0.170	1.105
$\alpha_{3}$	0.247	0.918

## Data Availability

Data is contained within the article.
